# Retracted: ‘We were on our knees long before COVID’: How healthcare workers experienced the compassionate care model during COVID‐19

**DOI:** 10.1002/capr.12545

**Published:** 2022-05-24

**Authors:** Rachael Kelleher, Lorraine McGurk, Sinéad Hannan, Charlotte E. Wilson

**Affiliations:** ^1^ School of Psychology Trinity College Dublin Dublin 2 Ireland; ^2^ Southern Trust, HSC Portadown UK; ^3^ Paediatric Psychology Service, Craigavon Hospital Southern Trust, HSC Craigavon UK; ^4^ Craigavon Area Hospital Craigavon UK

**Keywords:** compassion‐focussed therapy, compassion‐informed interventions, COVID‐19, healthcare workers, mental health, thematic analysis, trauma

## Abstract

**Aim:**

This qualitative study aimed to explore the impact of a novel compassion‐based intervention on healthcare workers during a pandemic.

**Sample:**

Participants were *N* = 10 healthcare workers (HCW) recruited from a healthcare organisation in Northern Ireland, including nurses, allied health professionals, managerial staff and administrative staff. The sample was 80% female with an average age of 45.1 years.

**Intervention:**

All participants engaged in a compassion‐based staff support psychological intervention. The ‘compassionate care’ intervention was based on the compassion‐focussed staff support model. Modalities included face‐to‐face, remote, individual and group intervention, offered by clinical psychologists and psychotherapists.

**Method:**

Ethical approval was obtained through the researcher’s academic institution. After obtaining informed consent, participants completed individual interviews, analysed using reflexive thematic analysis.

**Results:**

Findings highlighted model appropriateness and feasibility, detailing post‐intervention changes. Three themes were generated. The first two, *Preparing for war: A threat without boundary* and *Masking the pain: Externalising resistance to compassion,* describe the transition from an initial burnout state to a state of derealisation via avoidance coping. The final theme, *Reconnection: Returning to compassion,* highlights how participants rehabilitated via the intervention, reconnecting with relationships and personal values.

**Conclusion:**

Participants reported personal and professional experiential changes relating to compassion and resilience, while noting organisational blocks to compassion. The model appears appropriate across a variety of presentations and levels of chronicity of distress, across age, disciplines and gender groups, and for both managers and non‐managerial staff. Participants reported its usefulness in clinical and administrative settings, as well as application of the skills gained to settings outside work.

**Implications for Practice**

- This novel, brief compassion‐based psychological staff intervention appears feasible, well‐accepted and useful to healthcare workers (HCW), especially during COVID‐19.
- Healthcare workers noted the need for the intervention to be accessible; time, understaffing (not being able to leave their shift to attend) and not knowing about availability of the intervention were common barriers to accession in this study.
- According to the participants, skills gained post‐intervention included increased psychological resilience and improved emotion regulation skills; increased compassionate flow to self and others; promotion of adaptive health‐related behaviours like self‐care, hobbies and exercise; and increased confidence in their professional capacity. Many of these skills are generalisable to practice and use of skills was reported by staff across contexts, implying the versatility of the intervention.

**Implications for Policy**

- Overall, this novel, brief compassion‐based psychological intervention appears to promote and support staff resilience and alleviate the psychological distress associated with HCW trauma symptoms related to working during COVID‐19. The versatility of the skills and outcomes discussed qualitatively highlight the utility of such an intervention in healthcare organisational policy, for example in staff well‐being policy and educational curriculum.

## 1 INTRODUCTION

Compassion, as a concept, emerged from various philosophical, conceptual and spiritual and religious traditions such as altruism and Buddhism; thus, it has been variously defined (Gilbert, 2014). As a psychological construct, it has been defined by Gilbert as ‘a sensitivity to suffering in self and others, with a commitment to try to alleviate and prevent it’, a definition which is informed by Buddhist writings (ibid). As has been noted in the literature, there is somewhat of a conceptual overlap with mindfulness and coping. Disagreements about the definition of compassion exist, with some noting that the construct is somewhat controversial within the literature (for example, Goetz et al., 2010).

Compassion (which this paper will take to include self‐compassion) appears to be an important feature of alleviating transdiagnostic psychological distress (Wilson et al., 2019). It has been identified as such an important psychological construct to target therapeutically that it is a cornerstone of many third‐wave psychological therapies (Wilson et al., 2019), being included in the conceptualisation of their models or directly named in their models and interventions. Many of these therapies cite, as their theoretical basis for the development of compassion through mindfulness and meditation, from the Buddhist or Eastern philosophical tradition, including acceptance and commitment therapy (ACT) and dialectical behavioural therapy (DBT) (ibid; Gilbert et al., 2017). In ACT, compassion is not directly named as a key focus, but it is a value within the model’s concept of psychological well‐being (Neff & Tirch, 2013). Meanwhile, mindfulness‐based cognitive therapy (MBCT) (Segal et al., 2013) includes components which touch on self‐compassion (such as *metta* or loving‐kindness meditation, LKM) but with compassion as a secondary outcome. These therapies have identified the value of targeting the psychological construct directly or indirectly; indeed, there is much evidence that compassion is a key mechanism which affects the effectiveness of interventions (Neff & Tirch, 2013).

Meta‐analyses and randomised controlled trials have examined the impact of compassion‐focused therapy (CFT) and compassion‐informed interventions on a variety of clinical and non‐clinical populations (Kirby et al., 2017). In one such meta‐analysis, Kirby and colleagues found significant advantages of CFT and compassion‐informed interventions on change scores on several self‐report measures, including compassion (*d* = 0.55, *k* = 4, 95% CI [0.33–0.78]), depression (*d* = 0.64, *k* = 9, 95% CI [0.45–0.82]) and psychological distress (*d* = 0.47, *k* = 14, 95% CI [0.19–0.56]), and the results were noted to remain even when active control comparisons were included (ibid). Meanwhile, MacBeth and Gumley (2012) found a large effect size for the relationship between compassion and psychopathology, describing the construct as an explanatory variable in comprehending mental health and resilience. They describe compassion as an emotion regulation process and ‘motivational system designed to regulate negative affect through attuning to the feelings of self and others, and expressing and communicating feelings of warmth and safeness’ (ibid, p. 2).

Compassion also has some practical applications in a variety of directions in non‐clinical populations, such as in healthcare settings for staff. Compassion has been described by patients and families as necessary during healthcare procedures, with other types of literature identifying compassion as a patient right in healthcare settings and, thus, a component of quality care that should be regularly audited and reported on (Frampton et al., 2013; Paterson, 2011; Willis, 2015). Since the Francis inquiry into significant failings in patient care, many have argued that compassion should be central to the humanisation of health care (Francis, 2013; Jeffrey, 2016). Other literature asserts that compassion should be fundamental to various healthcare codes of ethics across healthcare professions, as well as being a necessary clinical competency for training and evaluation of healthcare work students and healthcare workers (Callwood et al., 2014; MacLean, 2014; Sinclair, Raffin‐Bouchal et al., 2017b; Sinclair, Russell et al., 2017a). Curtis et al. (2017) stated that self‐compassion is an important component in the provision of compassionate health care in the NHS and other organisations (also see Gilbert, 2009, 2013). Compassion may have important benefits not just for the self but organisationally, via staff health and quality of life, productivity and economic benefits (Cocker & Joss, 2016). Compassion can be argued to be fundamental to healthcare provision, because it appears to help healthcare workers (HCW) manage psychological distress. For example, Cocker and Joss (2016) noted that experiencing compassion appears to result in an increase in caring and altruistic behaviour, as well as fostering resiliency. Improvements in self‐compassion have been associated with a variety of positive sequelae relevant to this population, such as improvements in anxiety, depression and other psychological difficulties (Wilson et al., 2019). As such, compassion can be said to be an important construct to target within staff‐based psychological interventions, although Sinclair and colleagues note that the evidence base for compassion and its role in healthcare remains, to date, relatively less explored (Sinclair et al., 2016).

### 1.1 COVID‐19 and healthcare workers

Healthcare workers (HCW) are defined as ‘anyone who works in a healthcare or social care setting, including healthcare students on clinical placement, frontline healthcare workers and other healthcare workers not in direct patient contact’ (HSPC, 2021). Several studies have demonstrated the importance of occupational support for HCW, who have been noted to experience high levels of occupational stressors such as exposure to extreme illness and death (Sinclair, Russell et al., 2017a). In recent years, the psychological well‐being of HCW has been noted as a serious concern in the field of health care, with growing incidences of substance use, stress and burnout, and suicide and depression across all disciplines of HCW (Billings et al., 2021). Sendler and colleagues noted that prevalence rates for psychological difficulties are higher in HCW than in the non‐HCW population (2016). A body of cross‐sectional research suggests that this psychological distress relates to occupational factors of working with exposure to traumatic incidents such as illness and death (Wiederhold et al., 2018). Psychological distress and difficulties experienced by staff have wider service and economic impacts, as recent European workforce statistics estimate the cost of mental health difficulties (both work‐related and non‐work‐related) as being up to 240 billion euros per year (EU‐OSHA, 2015).

A unique stressor for HCW in recent years is the novel coronavirus disease. The coronavirus disease 2019 (hence referred to as COVID‐19 or COVID) was discovered in December 2019 in Wuhan, China (Fernandez et al., 2020). The disease was upgraded in status to being a pandemic by the World Health Organization (WHO) on 11 March 2020. Pandemics are defined by the WHO as ‘the worldwide spread of a new disease’ and by the International Epidemiology Association’s Dictionary of Epidemiology as affecting a ‘large quantity of people’ (Singer et al., 2021). COVID presented an unprecedented global crisis, with many complex variables troubling healthcare organisations including increased staff demand, increased exposure to medical trauma and global shortages of PPE (Livingston et al., 2020). Other large‐scale stressors of pandemics can include significant death rates, social and economic disruption (Madhav et al., 2018).

Research noted that, in the early stages of COVID‐19, HCW were reporting increased levels of psychological distress (Maiorano et al., 2020). In a systematic review of nurses' experiences of working during COVID, participants noted heightened levels of anxiety, fear of infection and death associated with frontline working directly with the novel infectious respiratory disease (Fernandez et al., 2020). Indeed, current research has strongly indicated the need for psychological interventions to support healthcare staff in stressful working conditions during the pandemic (Greenberg et al., 2020). As compassion has been suggested to be a helpful construct in improving a number of psychological sequelae (Kirby et al., 2017), such as the mitigation of fear, a compassion‐informed psychological intervention seems relevant in supporting the needs of HCW during a uniquely stressful and terrifying time.

### 1.2 The present intervention: Compassionate care model

One such therapy which directly purports to target self‐compassion is compassion‐focused therapy (Gilbert, 2009). While there are a variety of other compassion‐informed models, such as that conceptualised by Neff and Tirch (2013), a review of such models is beyond the scope of the current study, but has been summarised elsewhere (Kirby et al., 2017). According to Gilbert, Kirby and colleagues, CFT is notably differentiated from other compassion‐based interventions due to its theoretical underpinning, which is based in attachment theory, social mentality and theories of evolutionary psychology (in Kirby et al., 2017). The CFT model targets compassion directly through psychoeducation and behavioural components relating to the Threat, Drive and Soothe emotion regulatory systems, derived from an evolutionary model of compassion. Gilbert and colleagues propose three directions in which compassion is said to ‘flow’ or develop: (a) compassion for others, (b) compassion experienced from others to self, and (c) self‐compassion (Gilbert et al., 2017). In CFT, each of these directions can be a key focus for intervention; the three directions can also be interpreted as having a symbiotic relationship whereby the flow of compassion in one area can have related, adaptive changes in psychosocial well‐being. This study sought to investigate the impact of a psychological intervention on compassion in all three directions. The current intervention was based on the compassion‐focused staff support model (CFSS, developed by Dr. Kate Lucre of the Compassionate Mind Foundation). This model is based on the compassionate leadership model developed by West and Chowla (2017), based on the principles of CFT.

### 1.3 The present study

Healthcare workers can be more exposed to high levels of occupational stressors and trauma, particularly during a pandemic. As the recent COVID pandemic has been classified as a ‘a traumatic event of exceptional magnitude that transcends the range of normal human experience with exposure to risk of death’, it appears that HCW may need some significant psychosocial supports in coping with these stressors (Benfante et al., 2020; Dutheil et al., 2020). The unique factors associated with working in a high‐pressure healthcare environment, particularly during COVID, suggest a need to understand how a compassion‐based psychological intervention was experienced by HCW during this time. The current study aimed to explore these experiences.

## 2 METHOD

### 2.1 Design

As a qualitative study, participants were individually interviewed by the first author. Results were analysed using reflexive thematic analysis (RTA).

### 2.2 Sample

Participants were recruited from a healthcare organisation in Northern Ireland. Ten participants were recruited for the study, including *n* = 5 managers and *n* = 5 non‐managerial staff from various departments including the intensive care unit (ICU), emergency departments, specialist departments, social work and administration. Participants were from a variety of roles and disciplines, including frontline and clerical staff. The most commonly represented discipline was nursing, with seven participants from this discipline. The sample included both COVID‐facing and non‐COVID‐facing participants; on‐site, outpatient and remote workers were all represented in this sample.

Exclusion criteria, as described in the Information Sheet, included that the study contained questions relating to the participants' psychological well‐being, which may have caused some distress. This alerted participants to the possibility that, if they had pre‐existing mental health conditions, the study might be distressing to them and they had a right to decline participation.

Three participants had accessed the intervention originally for reasons not related to COVID (related to serious workplace events or stresses) but were recruited as either (a) their intervention overlapped with the onset of COVID in the organisation or (b) they reported using the skills while working during COVID. Three participants had also accessed eye movement desensitisation and reprocessing (EMDR) as part of their intervention.

The average age of the participants was 45 years and 1 month, and the age range was 30–59 years. The sample was 80% of female, with *n* = 2 male participants. Regarding participants' reasons for accessing the intervention, some initially accessed the psychological intervention for reasons not directly related to COVID (e.g. due to an organisationally related stressor); however, the majority accessed it for COVID‐related stressors. Note that, in Table 1 below, not all participants' demographic variables are displayed together for reasons of preserving anonymity.

**Table 1:** Participant demographic information

Participant	Pseudonym	Gender	COVID‐facing?	*N* sessions	Modality
001	Alma	F	N	2–3	Phone
002	Robyn	F	Y	Not known by participant: ‘several’	Face‐to‐face group, and individual phone
003	Catelynn	F	N	2	Face‐to‐face individual
004	Robert	M	Y	11	Face‐to‐face individual and phone
005	Olive	M	Y	1	Face‐to‐face group
006	Kayla	F	Y	1	Face‐to‐face individual
007	Emily	F	Y	2–3	Face‐to‐face group
008	Gabrielle	F	Y	6–8	Face‐to‐face and remote individual
009	Jacob	M	N	8	Face‐to‐face individual
010	Mona	F	Y	6–8	Face‐to‐face individual

### 2.3 Intervention

As can be seen in Table 1, participants could avail of a number of different therapeutic services as part of the compassionate care model used by the staff well‐being support team.

### 2.4 Referral process for intervention

The intervention was offered on a drop‐in basis or by scheduled appointment, if individuals or managers contacted the service using its dedicated phone number. The organisation’s communication team promoted the service via different mediums. Posters and flyers were placed in common areas in the organisation as well as on an online staff hub and app which staff could access at any time. All staff were contacted using a global email circular each week providing well‐being information, including the details of the staff well‐being support service. The service was further advertised through a series of interviews with the service lead, made available as podcasts and advertised by email and on the organisation’s social media platforms. All interventions were offered in as timely a manner as possible: the average length of time between referral and receiving the first episode of intervention was one week.

### 2.5 Format

On the organisation’s site, dedicated areas were allocated to the staff well‐being support team. A drop‐in service operated in a room onsite, a larger space which was decorated to provide a therapeutic ethos, used for group and individual sessions for both drop‐in and bookable appointments. Outreach sessions, available on request, took place in community‐based hubs off‐site within the wider geographical area of the organisation. Intervention was planned and tailored to the specific needs of the team or individuals requesting the service, and were typically requested by the team manager, who arranged staff attendance at group interventions. This facilitated timely use of the service and minimised potential impact on services and may have helped reduce barriers (geographical, travel, time, etc.) to accessing the intervention.

Participants could avail of the therapeutic intervention in a variety of forms, including face‐to‐face individual sessions, group and virtual sessions. For staff working remotely or on leave, sessions were facilitated remotely (by telephone or video conferencing software). The intervention was available on site on a face‐to‐face basis for the duration of the pandemic as it was determined that in‐person therapeutic intervention was an important part of the model. All social distancing and required infection control measures were adhered to while providing this service. Each session lasted 50 min, and were usually facilitated by a single member of the staff support team. The number of episodes of intervention attended by participants in this study ranged between 1 and 11 sessions. Many participants had their first engagement with the service by drop‐in, later returning for a more formalised set of 6–8 sessions. Other participants partook in a single or several drop‐in sessions.

### 2.6 Content

The intervention was based on the team’s ‘compassionate care’ model, developed by the second and third authors. This was a predominantly compassion‐informed model, with attention to different integrative individualised components such as trauma techniques, psychoeducation based on CFT and EMDR.^1^ It was devised from an understanding that the barriers that those in the caring professions may have to caring for themselves, namely asking for or needing care from others, may lead to shame and self‐criticism (Kirby & Gilbert, 2019). The model draws on both the compassionate leadership model (West & Chowla, 2017) and compassion‐focused staff support (CFSS, Lucre & Taylor, 2020), while also drawing on core principles of compassion‐focused therapy (Gilbert, 2009). The intervention content was based on a pilot study of the CFSS, which was developed and extended for local use (HSC, 2020; Tarleton et al., 2020). The key principles of the ‘compassionate care’ model are a visible and regular presence from the team; a warm, kind and non‐judgemental response to emotional distress; and sensitively noticing and responding to suffering using safe and effective therapeutic techniques to reduce, alleviate and prevent suffering. The model notes that staff in caring professions often do not seek support naturally, and that blocks to accession may include viewing help‐seeking as a sign of weakness (Kirby & Gilbert, 2019). The model also acknowledges that healthcare systems rely on carers to continue to provide care to others who are suffering, thus caring from others to support healthcare workers is essential.

In a pandemic, this emphasis was arguably more pronounced, with patients and staff suffering from the effects of the virus. Staff experienced a variety of stressors, like significant and prolonged health problems due to the virus, often leading to prolonged treatment, suffering and, sometimes, death. The model acknowledges the need to re‐engage the Soothe system in response to stressors, such as danger of infection and exposure to possible death, combined with the relentlessness of the working conditions, which can be said to constitute significant suffering for healthcare professionals. The intervention incorporated awareness‐building and skills‐based training to reduce activation of the Threat response and enhance the self‐soothing system, all embedded in compassion theory. These skills included grounding, breathing and mindful awareness, as well as CFT‐based psychoeducation of the three emotion regulation systems, for example noticing which system participants were currently engaged in. Key elements of compassion‐focused staff support were incorporated, with an emphasis on emotion regulation through connection, common humanity and compassionate action. In addition, trauma‐based components of non‐directive talking therapy were used to support HCW.

### 2.7 Organisational components

The staff well‐being support team acted as a ‘compassionate other’ on behalf of the organisation, incorporating ‘light touch’ elements such as maintaining a presence on wards for drop‐in appointments and building relationships with staff. The team modelled the key components of compassionate leadership in a top‐down manner, communicating compassionately with managers: firstly offering them compassionate care, then encouraging and advising them to respond to staff in a compassionate manner. The aim of the staff well‐being support team’s calm and visible presence was to normalise the concept of the ‘tricky brain’ response and to aid understanding of the Threat system reactions in the context of a new and complex global healthcare crisis.

### 2.8 Facilitation and supervision

The intervention was delivered primarily by qualified Senior and Consultant Clinical Psychologists who held Doctorates in Clinical Psychology. All were trained in compassion‐focused therapy. One facilitator was the research supervisor and third author of this study, and had worked with CFT since 2011; another was the model's co‐developer. The psychology staff support team consisted of psychologists and psychotherapists, and some of them were also trained in compassion‐focused staff support (CFSS). The CFSS model was based on Gilbert’s CFT model. The psychology team also offered compassion‐focused approaches as part of their core interventions in the organisation.

#### 2.8.1 Fidelity

One facilitator was a trained and experienced CFT therapist, with prior experience in delivering compassion‐focused staff support (CFSS) to healthcare staff in her role as Consultant Clinical Psychologist. This facilitator and service lead had supervision with the Compassionate Mind Foundation’s group supervisor and developer of the CFSS model for several years. Other facilitators were trained in delivering the Compassionate Care model and supervised by the service lead and model developer, who was always present during facilitation of groups to help increase fidelity to the model. The staff support team were also invited to attend a weekly therapeutic support group themselves over a 3‐month period, provided by the developer of the CFSS model, to allow them to experience the model for themselves. Regular supervision with the developer of the CFSS model, and on‐site supervision by the service leads, was provided. As the intervention is still in development, many components were not yet standardised, including length of intervention and whether participants received EMDR or not, which is a limitation of the study. However, all interventions were delivered with checks to increase fidelity under the CFSS model.

### 2.9 Ethics statement

Ethical approval was granted by the lead researcher’s educational institution. Further local ethical approval was not deemed necessary by Ethics, Research and Psychology Management staff in the organisation as the project was deemed a service evaluation. Participants engaged in a process of informed written consent prior to any participation.

### 2.10 Obtaining informed consent and verbal assent

Participants were given up to two weeks to consider participating in the study. The voluntary nature of participation was emphasised at all stages of recruitment and individuals could access the intervention without participation in the study. Informed written assent to participate was obtained before each interview; at the start of interviews, participants gave verbal consent recorded in the transcript as well as by signature where possible.

### 2.11 Recruitment strategy

All recruitment was virtual. Potential participants, who were previous recipients of the staff support intervention, were emailed the Information Sheet. Approximately 270 emails were sent, in response to which 15 participants expressed an interest in participating. *N* = 11 participants were interviewed. Two participants were excluded as they had not accessed the intervention. Another could not participate due to a COVID surge. The final two participants did not respond to the follow‐up email.

### 2.12 Interview protocol

Interviews were conducted remotely by video conference using the approved software or by telephone, and recorded for transcription. Interviews were conducted by the first author, who was not known to participants. Participants were given the option to leave their video off for the purpose of confidentiality and to reduce any burden associated with participation. Interviews followed a semi‐structured approach, guided by an interview protocol (Table 2) written by the researcher and supervisor. Interviews lasted between 35 min and 1 hr 5 min, with an average duration of 54 min.

**Table 2:** Interview schedule

What was it like working during COVID‐19? Did you know about the compassion‐focussed staff support programme at [location] before taking part? If so, what was your impression? What prompted you to access psychological staff support in the first place? What was it like for you to do the staff well‐being intervention with [staff member] and [staff member]? Sometimes in psychology we learn new ways of dealing with situations. What were the main skills that you learned during this group/talking to [staff member] one‐to‐one; this could be personal or professional examples? Which parts of the model in particular did you dislike/like? Did you notice any changes to how you experienced compassion during or after the intervention? If so, what were they? Were there any long‐ or short‐term changes for you? Do you notice any changes in how your peers or supervisors relate to you since the intervention? Do you have a similar or different experience to that?

### 2.13 Data management

Interviews were transcribed verbatim by the first author, with identifying names or events removed or changed. A selection of transcripts was shared with the research supervisor and clinical supervisor to check that anonymisation was sufficient and to examine bias in the interviews. All data were maintained by the researcher in accordance with General Data Protection Regulations (European Commission GDPR; Data Protection Act, 2018).

### 2.14 Analysis

The analysis was informed by a paradigm of interpretivism and constructivism, whereby the subjective meaning of the participants was co‐constructed by the researcher through dialogue and interpretation of the data (Ponterotto, 2005). A principle of analysis adopted by the researcher was to investigate participants' subjective experiences with a focus on participants' meaningfulness of experience and empowerment of the participants through this process (Elliott & Timulak, 2005). This was completed while taking into account any reflexive influences of the researcher’s own biases. Inductive reasoning informed the ‘open‐coding’ of the data (Braun & Clarke, 2013) within the framework of reflexive thematic analysis (RTA; Braun & Clarke, 2012, 2019). RTA has been described as a flexible approach to the interpretation of qualitative data, allowing for the derivation of themes and patterns (Braun & Clarke, 2012).

The following steps occurred iteratively over several months, particularly steps 3–6. There were three versions of theme development. For example, an initial four themes were developed: two pre‐intervention and two post‐intervention. Over time, these themes were deepened to reflect the abstract concepts to which some of the participants referred and to capture their lived experiences.

1. The lead researcher familiarised herself with the data throughout transcription and reading the transcripts. A reflective log was kept during this process, consisting of soft and hard copy notes and memos for reflection, as a memory aid and to raise awareness of repetition of codes in the data.
2. Initial codes were generated by the researcher based on the data. Based on the reflective process, the researcher tentatively held the initial codes as hypotheses while coding.
3. All data were coded line‐by‐line using these codes.
4. Codes were documented and grouped with similar codes or ideas, and themes were developed from these.
5. Potential themes were discussed with the final author. Initial themes were discussed as being more temporal and needing more narrative flow.
6. Arising from this discussion, the researcher reflected on the data and returned to the dataset for re‐coding, rereading and revision of themes. Potential themes were assessed across participants to gauge their relevance. The naming of themes and their narrative flow was considered by the lead researcher.
7. Themes were refined or regenerated until they were richer, more conceptual and a better fit for the data. Themes were collapsed or removed if they were not substantial enough in nature. From initial themes, reflexive themes were developed and a narrative was jointly conceptualised to describe them. This narrative was audited by the lead researcher and final author in an attempt to ensure themes were as relevant as possible.

### 2.15 Methodological rigour

Guidelines for quality developed by Yardley (2008) were used in the present study. These included robustly analysing the data, focusing on impact and importance, and being transparent about research‐related decisions. To ensure trustworthiness, several steps were taken as part of the research process (as explored by Connelly, 2016; Shenton, 2004). Literature discussion with the research team supported further elements of reflexivity. Resonation with the reader through the use of examples from the text was an important aspect of analysis, as was prudent flexibility of methodology, as discussed by Elliott and Timulak (2005). Credibility checks included comparison of the findings to the researcher’s results in the literature of their recently completed literature review on the impact of compassion‐based interventions for HCW (Kelleher, 2021). Other methods included immersion in the data (Byrne, 2021; Morrow, 2005) during the analysis stage so as to ensure as adequate an interpretation of the data as possible.

## 3 RESULTS

Three themes were generated. The first two themes, *Preparing for war: A threat without boundary* and *Masking the pain: Externalising resistance to compassion*, describe the transition from an initial burnout state (characterised by persistent stress and feelings of overwhelm) to a state of trauma and derealisation via avoidance coping. The final theme, *Reconnection: Returning to compassion,* highlights how participants used the intervention to rehabilitate their adaptive and affective functioning via reconnection with their interpersonal relationships and personal values.

### 3.1 Theme 1: Preparing for war: A threat without boundary

In describing the state of healthcare working pre‐COVID, participants communicated a level of utter depletion personally and professionally, such that COVID appeared to be the final ‘straw’ (Mona). There was a reported experience of trying to engage with an invisible enemy, with several using war metaphors to describe the onset of the pandemic. Perhaps, for a group of individuals who frequently referenced being soothed by order and ‘control’ (Alma), one of the insidious components of COVID for participants may have been the lack of boundary to the threat, as the pandemic was transmitted rapidly and internationally in an airborne manner. Participants described trying to use old strategies to cope with the invisible yet very real threat of the disease, but finding these ineffective in managing the patient surges and associated occupational stress, they appeared to find it difficult to motivate themselves and their colleagues to ‘keep going’ (Gabrielle) and to retain mindful awareness to see past the worst of the pandemic to the future (Emily). Familiar strategies participants might have used for replenishing psychologically to mitigate burnout and reconnect with the true self (time off, holidays, spending time with loved ones, hobbies, relaxing by enjoying media) were unavailable to them during COVID and most spoke of the difficulty of this. This appears to be in line with Gilbert’s CFT conceptualisation of the need to Soothe through interpersonal connection and compassionate self‐self relating to manage the deleterious effects of the Threat system (Gilbert, 2014). Due to staff illness and shortages, Emily described how all annual leave was cancelled for staff in the state of emergency during the COVID surges, leading to a state of exhaustion amongst her colleagues. Over the course of the pandemic, ongoing at the time of writing this study, participants described various states of emotional exhaustion and derealisation in the absence of such supports (Kayla, Emily). It appeared that the sustained nature of the threat, and the surreality of the pandemic, may have led some participants to question the nature of their reality‐building strategies and, on a deeper level, their values and identity (sub‐theme 1.C). It is possible (based on derealisation, fear and the unique stressors) that participants worried about an erosion of their own compassion when they spoke of blocks to compassion they experienced during COVID (Theme 2).

### 3.2 Sub‐theme 1.A. Managing the unmanageable

This theme encapsulates the pressures of healthcare work before COVID. Many participants noted the pressure of healthcare work before onset of the pandemic, with reportedly stressful working conditions, difficulty communicating with management, high professional standards and the feeling that management did not appreciate the difficulties of the HCW environment (Robyn, Robert and Mona).

Emily described how many HCW she knew had retired or left the profession in recent months since the onset of COVID, while Kayla described changing roles as she felt underappreciated for her work efforts. Mona put it most starkly, stating, ‘We were on our knees long before COVID’. The use of the word ‘we’ implies a collective sense of burnout experienced by her profession. The use of the colloquial phrase ‘on our knees’ implies a deeper sense of vicarious trauma pre‐COVID that was perhaps more viscerally experienced by Mona and her colleagues. Robert reflected a similar sentiment, emphasising that, ‘I have seen things that would make your hair stand on *end*’.

Many noted high levels of professional perfectionism within their specialisation. There was much agreement amongst ICU, frontline and nursing staff regarding professional expectations within their peer group, in particular, ideas of ‘perfect’ workplace performance in a situation where mistakes can lead to patient injury or death:

‘If you’ve ever met any [profession] we’re very much in control, and kind of nearly take *pride* in being in control…I have very, very high expectations of myself, and I expect those expectations of others…in [work] if you make a mistake someone dies… If you do something wrong, someone dies…there is an expectation that you must, and *should,* be nothing but perfect’. (Robert)

‘You sorta expect an awful lot of yourself you know? You strive to just be perfect…for the patient…and control everything to make sure that that patient has the best chance. So there’s certain parameters and certain levels of care that we expect of each other and of ourselves’. (Gabrielle)

‘We’re in a business that cares for other *people*…that is at the forefront of what we do…trying to deliver for our service users, and …not about thinking about ourselves’. (Catelynn)

Emily described a member of staff trying to work,

‘nearly in a *collapsed state*…shouldn’t have been *driving* even and I thought ‐ if that was a member of the public [they] would’ve gone straight to casualty; [staff] just felt that [they] wanted to get back to work… (*crying*)… you seen staff come in [*sic*] very, very ill, wanting to keep working… but they were trying to get in and help and be fine…go back to work… they’re very dedicated people’.

### 3.3 Sub‐theme 1.B. Getting ready for battle

Many participants described their experience of COVID in stages, with preparation being a precursor to the following sub‐themes of trauma responses. Participants' initial psychological response to such unique stressors appeared to be high levels of anxiety in preparation for the onset of the pandemic, with staff being advised to take measures they had not yet experienced in their careers to date. Gabrielle described the anticipatory anxiety of preparing for COVID to ‘hit our shores’, while Robert described his fear in reaction to the drastic measures taken in preparation:

‘Overnight, there were Perspex screens nailed floor‐to‐ceiling and blocking out that whole patient space…it was *shocking* and anxiety‐inducing, and you realize that the level that we’re having to go to; it was something that you would see in…an outbreak movie’.

Meanwhile Emily noted the process was akin to ‘getting ready for *war,* you’re preparing the troops; your colleagues’.

### 3.4 Sub‐theme 1.C. Derealisation: Dissolution of boundaries and values

After the initial COVID surges in the healthcare organisation, participants described the overwhelming nature of the constant bereavement they experienced while they were working with the disease. Mona, Kayla, Gabrielle, Robert and other participants described a strong feeling of grief and overwhelm, despite having extensive experience in medical training and preparation for emergency settings. Emily described the COVID‐related scenes she witnessed:

‘Putting them in body bags and they were dying…one after the other after the other’.

The repetition of her words may reflect the repeated experience of numerous deaths due to COVID, and the imagery she used in her description may suggest she retains mental images of this task. The trauma of COVID was described as uniquely complex and more emotionally taxing than participants' previous experiences of difficult work‐related stressors. Words used to describe the horror of what participants had witnessed included ‘horrendous’ (Robert, Mona), ‘awful’ (Emily), and like a ‘fog’ (Gabrielle) or ‘blurred’ (Robyn), with others referencing unspeakable elements to the trauma: ‘The whole year…I just wanna put it away and forget about it’ (Robyn).

Additionally, participants described trauma related to bereavement following the deaths of patients they cared for, including a number of suicides or deaths of their colleagues who were HCW during COVID (names redacted for confidentiality).

Gabrielle ‘The sheer loss (*sighs*) I don’t think I’ll *ever* get over it because…it wasn’t like normal loss that you have’.

This appeared to lead to a strong sense of surreality, sometimes dissociation, in the participants:

‘You felt a bit more disconnected from your patients’. (Gabrielle)

‘I just wasn’t *engaged* ‐ I was hazed…I was just so tired and I didn’t want to talk to anyone’. (Robert)

It was often at a tipping point that participants realised the extent of their dissociation, which might have been ongoing for some time. Gabrielle described this point as, ‘I just fell apart…I knew*’*. Frequently, this tipping point was a trigger for their referral to the staff support service. Kayla described her tipping point occurring when she experienced an episode of dissociation when caring for a patient:

It was the first time in a long time I’d ever felt *disconnected (sighs)*…[patient] obviously was on ventilators… it was almost like he was…. a *machine* that needed to be fixed rather than an actual human *being…* I was just like “This isn’t the [type of work] that I want to do”'.

Here, Kayla referenced a connection with her personal values of her own standard of professional care, which she was aware was becoming impacted by her COVID‐related distress. After this episode of dissociation, Kayla reported commencing psychological intervention and a period of leave as she realised she could not ‘settle’ her worries about the threat of COVID to her child, explaining how ‘alarm bells started going off in my head’. Others reported a similar experience (Robert, Mona and Gabrielle), describing disconnection from their personal values due to high levels of overwhelm and dissociation. This dissociation was described physically and metaphorically by Robert when he stated, ‘trying to re‐identify who I was when I looked at the mirror’, and in relation to psychological distance from one’s own values and self:

‘It goes against everything that you’ve been trained for years and years…our COVID patients just blew [our quality of care standards] out of the water because we were having to lower our standards’. (Gabrielle)

Like many others, Gabrielle described that her personal relationships were impacted as a result of the ‘pressure’ of working in such extreme conditions: ‘You could feel the anger and frustration. And *I* could feel it more…I know I got sharper’. Gabrielle and others noted having limited patience with other colleagues, as they became increasingly emotionally drained throughout COVID. Mona stated: ‘I *always* was pleasant to people…I noticed that before I went [on leave], I was getting *ratty*, and that’s *not me*. It *never* was me’.

This theme impacted the second macro theme, as blocks to compassion appeared to arise from this traumatic context and from disconnection to others, whereby participants described an extreme oscillation from the affective and physiological states of suspense to panic.

### 3.5 Theme 2: ‘Masking’ the pain: Externalising blocks to compassion

Blocks or resistance to compassion was reported by most of the participants in this study. This theme will discuss the potential origin of these blocks (sociocultural components) and the extent to which the various blocks may interact (organisational to relational). In line with the three directions of ‘compassionate flow’ described by Gilbert, these blocks appeared to be relational in nature; intrapersonal (self‐self); interpersonal (self‐other); and external (other‐self). Mona described the relational component to this block as having ‘a *mask* on and they’re…all jolly and stuff but they feel like *crap* inside. I’ve been there, I’ve been in that spot’.

Participants described a sense of watchful waiting and later derealisation in relation to the COVID crisis and related protocols. It may be that while attempting to survive in a traumatic situation, higher order, non‐vital cognitive functions such as compassion, perspective‐taking, mindful awareness and rationalisation of threats were deprioritised by participants (as suggested by Porges, 2009). This type of sustained threat may have led to the externalisation of internal distress directed towards tangible objects or extant organisational structures, after the initial period of forming a group in solidarity against the disease: ‘you always seemed to be fighting against *somebody else*, while trying to do your job. Whereas the first wave was you were allowed to do your job’ (Gabrielle).

Indeed, several blocks described by participants were practical or physical; not being able to see patients or colleagues, or difficulty connecting with psychology staff due to COVID health protocols like personal protective equipment which staff were required to wear (Gabrielle and Emily) and social distancing protocols like virtual meetings (Jacob). These practical blocks may have served as a metaphor for the relational ‘mask[s]’ Mona referred to earlier. Medical masks, visors and gowns potentially act as physical barriers to reconnection with other people, due to the impact on important aspects of social communication including eye contact, facial expression and body language (Frith, 2009). As suggested by the evolutionary‐based models of compassion, and trauma and comparative psychological literature, reconnection with others through interpersonal expression of compassion, affiliation and warmth after adverse experiences may be an important part of the emotional regulation of distress, as well as enabling the assimilation and processing of the events (Bell et al., 2017; De Waal, 2008; Gilbert, 2014).

### 3.6 Sub‐theme 2.A. Saturation of external stimuli

In the initial months of COVID during the global and regional restrictions known as ‘lockdowns’, many reported a pervasive sense of uncertainty and high levels of stress which appeared linked with increased psychological responses of stress, depression or anxiety; even in those without previous history of psychological difficulties (Kelly, 2020; Shah et al., 2021). This was reflected in participants' experiences while working in a designated COVID healthcare organisation, where they were saturated by high patient numbers, busy and noisy working conditions, and discussions about COVID.

Many participants reported feeling over‐stimulated by COVID‐related media content. Some handled this by avoiding COVID‐related information completely, by switching off medical dramas or the news, or avoiding discussion with family and friends about the pandemic (Kayla and Gabrielle):

‘Everything just revolved around the pandemic. Everything. Everything at work, at home, everywhere just revolved around the pandemic…I always hated too, on your time off they were always asking you about work. And I was like, “I have very little time off. *Please* stop. I don’t want to talk about it.”’ (Robyn)

‘they had COVID episodes in *[favourite TV show]*..you realize—everything nearly came flying back…you kinda just felt‐ very *anxious* and the *anxiety*—and it was only *then* it kind of hit you’. (Olive)

Others felt consumed by COVID‐related media and official guidance, describing that they could not ‘switch off’ after work, with Jacob stating he was ‘glued to the news’ at night and Robyn noting that she could not ‘get my mind to switch off—couldn’t actually go *home’*.

With no traffic on the roads and only necessary retail open, the media reported an eerie sense of quiet; indeed, there were significant reductions in noise pollution in Ireland (Basu et al., 2021). In sharp contrast to this, the only places of activity globally were hospitals and healthcare organisations. While working in these busy healthcare organisations, participants reported a variety of psychological and physiological symptoms during the onset of the COVID epidemic. These included increased blood pressure, insomnia, irritability, tearfulness, panic attacks, return of old psychological difficulties, worsening of chronic health conditions, anger, fear and frustration. While many described symptoms including depersonalisation, dissociation and an activated fight‐or‐flight response that appeared sensitive to any threat‐based stimuli (Kayla), Robert explicitly mentioned concerns about he and his colleagues developing post‐traumatic stress disorder.

### 3.7 Sub‐theme 2.B. Sociocultural blocks to engagement

Participants reported a range of resistance or avoidance behaviours towards the intervention. Reasons commonly cited for not accessing psychological support included the following: feeling that they did not need it; worrying that it would be a waste of time due to using the resources intended for another person; or that they did not need self‐compassion in general. These beliefs were usually described in relation to another person’s experience; for example, participants compared their experiences to other staff with other personally difficult circumstances. The exceptions to this finding were Olive (P5), although she noted she could be hard on herself in work; Mona (P10), who noted that she was ready to receive compassion; and Robyn, who noted she was open about her attendance in the intervention and was keen to engage during sessions.

An important part of the reported blocks to compassion appeared to be the sociocultural context of participants' professions (as in Theme 1). Participants noted that their colleagues appeared to be resistant to self‐compassion and psychological interventions, which appeared linked to an impression of professionalism. Similar to literature that has previously documented traits of conscientiousness and perfectionism within healthcare workers (Pérez‐Fuentes et al., 2019), many noted that a key part of their experience of high professional standards was not being seen to need psychological support (Robert, Alma, Catelynn, Robyn):

‘you think it’s a weakness ‐ It’s not really… I think people put that pressure ‐ you put that pressure on yourself because people expect a [profession] to be… whatever. I just needed permission to go. I just needed someone to give me permission to go, to *tell* me to go. Even though I knew deep down I had to take time off. I just needed somebody to say “*You* need to go”’ (Gabrielle)

Related to this, many noted a fear or feeling they were letting staff down, which conflicted with their own need to take leave:

#x02018;we already were short staffed, the [profession], there were so many less, and you’re just thinking, like, “If I’m not there, who’s going to be there?”’ (Gabrielle)

Several participants (Alma, Robert and Olive) noted that their colleagues appeared to be intimidated by the label ‘psychology service’, or to feel that they did not need psychological support during COVID:

‘For some, the word psychology is a scary one that means “I’m not well”, instead of seeing it as an opportunity to keep healthy’. (Alma)

An important sociocultural system in this service evaluation was the Northern Irish context. Specifically related to this, a number of participants noted that they felt the public did *not* show them compassion. They noted experiences such as their friends not believing in COVID and not understanding their experience (Robyn); friends or family forgetting to ask about their well‐being; and members of the public being verbally aggressive in communication (Catelynn). Alma described feeling deflated by an experience of witnessing a lack of compassion within the public sphere, while in a supermarket with little food left on the shelves and witnessing frenzied consumer behaviour, with customers ‘pushing’ an ‘elderly person’ present. Gabrielle described the pain of viewing news coverage regarding the COVID surges in Northern Ireland as a ‘knife in the back’ as she felt it represented the sentiment that ‘the support from the country was completely… gone’.

Cultural blocks to compassion also included the difficulty of participants expressing themselves emotionally, which some hypothesised may have been related to the Northern Irish context in which they lived. For example, Robyn noted ‘My [parent] would never really *understand* emotion and I was never really brought up to show it or talk about it…I don’t know wherever the North are a wee bit poorer about talking about how they feel as a whole’. Gabrielle also described keeping a ‘guard up about yourself…that’s from childhood, to protect yourself’.

### 3.8 Sub‐theme 2.C. Organisational blocks to compassion

The participants of the present study reported feeling increased pressure from internal and external sources. Participants noted pressure related to different expectations for their roles in work; in particular, new organisational responsibilities were experienced by participants during the pandemic through redeployment or promotion to a higher level of responsibility. Gabrielle noted that she was not financially compensated for this increased responsibility, leading to her feelings of frustration, upset and anger.

The theme of forgetting compassion was also strongly reported across participants within an organisational context. Managers noted staff feeling misunderstood by their managers and vice versa (Alma). Robyn and Robert described staff shouting at each other; Mona noted an episode of feeling publicly humiliated by a colleague; and Gabrielle described feeling demoralised by negative feedback from quality control during COVID. Emily and Kayla felt that their leave or time off was not respected, as both discussed how they were contacted by their organisation while on sick leave. Jacob and Catelynn noticed difficult workplace events had impacted the compassionate flow within their teams, with initial shock and disconnection, and, later, feelings of reconnection post‐intervention. Robert, Mona and Kayla voiced strong concerns about the blocks to compassion from a managerial and systems level towards staff, associated with their feelings of not feeling valued by the organisation.

Many others noted a block to compassion was not having the time to access the intervention (Emily, Gabrielle), including feelings of guilt that they would be leaving their colleagues with more work to do if they attended the sessions. Others noted blocks including not knowing about the intervention’s availability (such as Jacob). The intervention was often promoted by email, but many noted not even having time to check this (Mona, Gabrielle).

### 3.9 Theme 3: Reconnection: Returning to compassion

Despite these reported blocks and their traumatic experiences, participants were able use the intervention to reconnect. This highlights an important aspect of the findings, that, despite feeling emotionally drained and sometimes fearful of experiencing the ‘traumatic’ work of processing aversive emotions in the psychology intervention (Robert), participants still attended the intervention and engaged with the content. They continued to engage with compassion on a daily basis, despite ostensibly already being under strain in their role as HCW for the NHS, the post‐traumatic stress of living in a politically tense environment, intergenerational trauma associated with the troubles in Northern Ireland (Campbell et al., 2004; Fitzgerald et al., 2017), and experiencing the emotional isolation of working alongside other stressed HCW (described by Olive as ‘loneliness’). Arising from their attendance, they appeared to gain skills which they reported leading to increased resilience, awareness and confidence in their work and themselves. Participants described a movement away from the apparent over‐activation of the sympathetic response towards adaptive states of grounding and re‐orienting to reality.

This appeared to occur via several therapeutic processes including skill acquisition, increased self‐reported resilience, increased mindful awareness of emotions and increased confidence.

### 3.10 Sub‐theme 3.A. A unique space

Participants noted that speaking to their colleagues worsened their distress, perhaps as it appeared to expand the perception of the threat:

‘When I talked to my colleagues about [COVID], it was a *negative* experience… It wasn’t a helpful experience because all it did was, you know, reiterated that we don’t have support, that I don’t have the time, that I’m too tired, that it’s getting worse and there’s no end in sight’. (Robert)

Robert, Olive, Jacob and Gabrielle noted that the service was more beneficial to them than speaking to colleagues or friends and family for support. Participants also noted that the staff support service provided them with a different type of support to other modalities offered within the organisation, describing the intervention as being different and more beneficial to them than their experiences of Occupational Health, the internal organisational telephone support, the organisation’s online staff hub, and clinical supervision (Mona, Emily, Robert and Jacob). Olive described the staff support intervention as, ‘Somewhere that I wouldn’t have had before’, while Emily noted, ‘you could really talk out what you—what happened, you know, what you’ve seen and witnessed, or how your feelings are just on that one day…you’re being cared for instead (*pauses, sobbing)…’* **.** Catelynn, Robert, Gabrielle and Emily expressed concerns about the service’s continued existence, stressing that they felt the intervention should continue even as COVID surges waned. Several participants described a ‘fantastic’ experience (Catelynn) with the service: ‘[Psychologist] *wanted* to *help staff*…[she/he] knew that we needed help. I think [psychologist] felt that [she/he] wanted to establish something that would help staff’ (Gabrielle).

Prior to the intervention, participants noted the impact of COVID and related adverse events (including deaths and stressful organisational events) that had led to feelings of anxiety, panic, loss of confidence, dissociation and stress. Some participants noted post‐intervention feelings of increased calm and mindful presence in everyday life. Such feelings were apparently related to particular skills they had acquired, including breathing techniques, grounding, mindful awareness, self‐care skills, psychoeducation, normalisation of their experience, understanding the need for self‐compassion, reduced self‐criticism and self‐punitive thoughts/actions, as well as creating and maintaining mental boundaries in relation to COVID and work. Recognition of transferential feelings and normalisation of experience was mentioned by a few participants. Olive, Catelynn, Robyn and Mona described awareness of which emotion regulation system they were in, and learning that life events could impact the Threat, Drive and Soothe systems:

‘all the stuff that [Psychologist] did, [he/she] *really* opened my eyes and my mind to *why* I possibly might *feel* this way’. (Mona)

A reduction of guilt or shame around their feelings was reported following psychoeducation on emotions, with many reporting an array of changes after the intervention in relation to their self‐concept and self‐esteem. Many noted changes to their priorities, beginning to implement changes regarding what was important to them (including hobbies, friends, family and alone time).

Other skills reported by participants related to their occupational functioning within the organisation, including an increased ability to delegate (Olive), reduction of self‐blame in relation to work errors or work‐related events (Mona), restored ability to prioritise, restored performance in relation to specific work tasks, increased awareness of burnout and self‐monitoring of when they needed to take breaks or annual leave (Robert and Alma). Many noted that these changes were longer term, although some noted changes which were shorter term.

Participants reported surprise at the transferability of skills to a variety of environments including home and work (e.g. Olive and Kayla reported using their skills outside and inside work). Some participants noted that increased self‐compassion subsequently increased self‐reported workplace productivity and confidence, prioritising own needs and mental health. Olive noted: ‘you were a wee bit more *confident* again…you’re just back to how you were *before*’.

### 3.11 Sub‐theme 3.B. Increased compassion in three directions

The theme of more nuanced and developed compassion was cited by many of the participants in the study. This compassion was mentioned in relation to self‐to‐self relating, self‐other relating and other‐self relating. Several noted an increase in self‐compassion post‐intervention, by consciously changing their behaviours to more self‐compassionate actions, or relating to their thoughts in a more self‐compassionate way. Several realised the value of compassion over the course of the intervention and after it ended, exemplified by Olive’s observation that ‘you can’t pour from an empty glass’.

Participants also were more able to notice compassion from others towards themselves. This was experienced in person and virtually, as well as through gifts from other organisations towards HCW (food, toiletries), acts of service and other public acts of recognition of the work of HCW. Emily noted the recognition of her work by the public: ‘There was great kindness from the *public…* People were coming out to clap…(*crying*)—it was lovely to hear them…people appreciated what you were doing’.

Staff reported feeling more motivated to be compassionate to their colleagues, by supporting or encouraging them to access the intervention (Emily and Robert) and by being more mindful of colleagues' well‐being (Jacob). Catelynn noted making greater allowances for colleagues or members of the public, as she noted increased perspective‐taking capacity towards others post‐intervention.

Increased collective compassion and optimism was explored by many participants. Emily and Jason reported the positive aspects of working during COVID, including having ‘fun’ when redeployed and meeting new teams (Emily) and the enjoyment of remote working. Others (Robert and Olive) described the uniquely bonding experience of a feeling of connecting with their colleagues while working during a stressful time.

## 4 DISCUSSION

This study aimed to explore the qualitative impact of a novel, compassion‐based psychological intervention on HCW in Northern Ireland during a pandemic. *N* = 10 participants from Northern Ireland were interviewed and RTA generated three themes: (a) *Preparing for war: A threat without boundary;* (b) *Masking the pain: Externalising resistance to compassion;* and (c) *Reconnection: Returning to compassion*. Themes were all narratively interrelated and demonstrated the participants' temporal perspective change from trauma, and some maladaptive coping and stress pre‐intervention to compassion, adaptive emotion regulatory responses and increased awareness of affect post‐intervention. The intervention helped participants to experience compassion in three directions (to self, from others and to others), which appeared to be a valuable resource in coping with their psychological distress. The intervention was feasible, well‐accepted and useful to the participants of this study, especially during COVID‐19, although organisational blocks to compassion could ensue. Participants reported that these need to be dealt with as part of the ongoing systemic component of the intervention.

Like many HCW during COVID‐19 (Sheraton et al., 2020), participants described initial trauma‐type responses to working during the pandemic, especially frontline working. The dissolution of typical social structures (working from home, national states of emergency declared, redeployment and unfamiliarity with new social and healthcare protocols) may have led to a form of collective boundary dissolution whereby defence mechanisms or maladaptive coping behaviours are activated to regulate affect (Marčinko et al., 2020). Such coping mechanisms may include fear, anger and confusion (Kelly, 2020), or derealisation and numbing (Subtheme 1.C). The unspeakable nature of the trauma, reflected in the theme of *Saturation,* is supported by literature on post‐traumatic stress disorder (PTSD) symptomology regarding alexithymia and hypersensitivity to trauma‐focused stimuli (Bell et al., 2017).

Additionally, participants reported personal barriers to compassion which complicated their ability to self‐soothe in the context of trauma. The theme of masks or personal blocks to compassion (discussed in themes 1 and 2) appeared mostly transformed post‐intervention. However, organisational blocks to compassion remained an issue for many participants like Robert and Kayla. It appeared that participants' perspectives towards these blocks had softened or been contextualised via perspective‐taking, with Mona describing a positive re‐appraisal of being ‘just a number’ within a large staff body, and others describing a reconnection with their personal values post‐intervention (Theme 3). However, an important aspect of compassionate leadership in organisations arguably involves the recognition of unique stressors in healthcare working (de Zulueta, 2016), made more complex during COVID‐19. An important finding from the current research is that, while the intervention appears to have assisted those HCW with psychological distress, such an intervention must be scaffolded by compassionate values within healthcare organisations (supported by Curtis et al., 2017).

Participants noted the difficulty of working with these organisational blocks in the form of fundamentally unchanged aspects of the organisation. Interestingly, it appeared that the intervention somewhat mitigated the organisational and sociocultural blocks to self‐to‐self compassion. This was perhaps due to skills gained, including increased psychological resilience and improved emotion regulation; increased compassionate flow to self and others; promotion of adaptive health‐related behaviours like self‐care, hobbies and exercise; and increased confidence in participants' professional capacity (Theme 3). Many of these skills are generalisable to practice, and use of skills was reported by staff across contexts, supporting the versatility of the intervention. Indeed, the constructive nature of experiencing compassion to others is supported by recent research which suggests that HCW can experience mental well‐being improvements via compassionate relationships with service users (Ortega‐Galán et al., 2021). Certainly, the results of the current study emphasise the vital nature of compassionate relationships in sustaining the psychological well‐being of HCW during a uniquely stressful time.

### 4.1 Strengths

This appears to be the first study known to the researcher to use the CFSS model with healthcare workers in a COVID‐designated healthcare organisation during the pandemic. Fidelity to the model was enhanced with supervision from the founder of the Compassionate Mind Foundation. The study may also be said to demonstrate cross‐model integration as participants applied mindfulness components to their clinical practice in frontline and non‐frontline healthcare work. This may have been completed through mindfulness and acceptance‐based components, such as accepting intrusive thoughts, and compassion components such as enacting the Soothe system of the CFT model. The study also benefited from the intervention being enacted systemically and developed based on the feedback and needs of HCW. The sample size was appropriate for qualitative research, based on a previously cited recommendation of 5–25 participants (Creswell, 1998). The sample was as representative as possible of the organisation’s workforce, with a range of disciplines and levels of seniority included. The sample represented various attitudes towards attending, from overt resistance or fears of attending, to those who had attended prior psychological interventions or who had positive appraisals of such interventions prior to taking part in the intervention. Interviews were semi‐structured, allowing for the researcher to follow lines of enquiry which were more individualised to each participant while adhering to a basic structure of questions asked to all participants, potentially capturing greater nuance and richness of participants’ values and overall themes within the specific sociocultural context of Northern Ireland (a strength of qualitative research; Choy, 2014).

### 4.2 Limitations

The current study has a number of limitations. As this is an exploratory study of a novel intervention, many components of the intervention were not yet standardised. While this was a qualitative study without the need for a control, some form of comparison would have been interesting. Other confounding factors in terms of fidelity to the model under investigation in the present study included some participants also receiving EMDR as an intervention, the intervention being delivered by different members of staff and non‐standardised length of intervention. Methodological limitations may include the use of a non‐standardised interview protocol which may have led to bias in questions, a small sample size, and single‐site recruitment via convenience sampling.

The researcher did not collect sufficient demographic data to comment on demographic variables such as race and ethnicity, another limitation of the study. This sample was not gender balanced, although this may represent the gender balance of healthcare workers as WHO statistics suggest that 70% of the global healthcare workforce identify as female (WHO, 2021). Indeed, the impact of gender and length of time in participants' respective professions are two variables which may have impacted the findings and were not explored in the present study. Quantitative measures would have added more robustness to the findings but were beyond the scope of the current investigation. Psychometrics to assess adherence to the model could be useful. Another limitation of the current study’s research design is the difficulty ascertaining objectivity of the findings. For example, an important theme may have been overlooked, and thus, the researcher’s skill at interviewing may have directly impacted the results (Choy, 2014). This type of design is also far more time‐consuming and perhaps less replicable by HCW who wish to complete service evaluation using a similar design (Choy, 2014; Queirós et al., 2017).

### 4.3 Implications

Regarding practice implications, HCW in this study highlighted the need for the intervention to be accessible. Time, understaffing (not being able to leave their shift to attend) and not knowing about availability of the intervention were common barriers to accession, discussed in Theme 2. Areas for change or improvement were reported to the intervention’s developers and should inform the ongoing provision of this intervention for HCW in this healthcare service. The findings could be generalisable to other compassion‐based interventions, particularly those conducted in a healthcare setting. It would be interesting to see this study replicated in other sites and countries in order to determine the extent to which some of the results may be culturally bound, while other findings may be salient across a variety of sociocultural contexts.

The rich literature on COVID, which is still emerging, should inform any ongoing research into psychological interventions for HCW due to some of the unique stressors associated with healthcare working during a pandemic, as described by the participants of this study. Directions for future research could also include the introduction of more ‘light’ psychoeducation, as was included in this study, for example through the presence of psychology staff in healthcare sites throughout the week, or in a smartphone application as suggested by Finlay‐Jones et al. (2015). As many participants mentioned finding time to practice skills as a block to compassion, a useful app feature could be daily reminders and prompts via syncing with other smart devices, like smart watches, regarding skills‐based techniques like diaphragmatic breathing or compassionate awareness. As was suggested by some participants, like Alma and Catelynn, positive role modelling may help reduce some barriers to accession of the service, through the provision of managerial or other participant testimonies of those who have taken part in the intervention. The use of psychometrics could also be incorporated to provide longitudinal feedback data over time to assess some of the symptoms the participants described in this study, including dissociation, anhedonia or loss of confidence.

These recommendations are suggested to support the key implications of this research, namely that compassion‐based interventions for HCW experiencing distress appear a vital resource during the pandemic, and the organisational scaffolding and further development of such interventions are strongly desired by the participants of this study.

## 5 CONCLUSION

The COVID‐19 crisis has highlighted the negative psychological sequelae of pandemics, especially for HCW. It has increased global awareness that rather than being a remote historical threat, pandemics may be an ongoing inevitability. In the context of a post‐COVID world, particular attention is merited to qualitative findings on developing psychological support for HCW who have experienced psychological distress related to COVID, and for those who may do so during future pandemics.

Results of this study revealed three key findings: (a) HCW experienced significant ongoing PTSD‐like symptoms and psychological distress in relation to working with COVID; (b) this distress appeared to be alleviated by a novel, compassion‐based brief psychological intervention; and (c) participants noted the need for expanded and continued psychological support as they continued to work through COVID surges and post‐COVID.

Findings suggest future areas for research, like an expansion of psychological staff support services and a possible replication of the intervention to assess effectiveness and further develop the intervention. Such compassion‐based interventions could seek to explicitly target HCW, especially those with more self‐sacrificing tendencies or more complex resistances to compassion, and could draw on the extant PTSD literature as this was a large part of the symptoms reported by participants in this intervention. Developing compassion‐based interventions for essential staff (HCW) within a time‐poor working environment such as healthcare should be a priority in the midst of, and the eventual wake of, COVID‐19. The reconnection to compassion appears to have been a fundamental lifeline to the participants of this study working in the context of a pandemic and the associated trauma that they reported experiencing. To best support these essential workers through a period of extreme strain and psychological vulnerability, healthcare organisations must recognise their duty in compassionately supporting their staff.

## CONFLICT OF INTEREST

No potential conflict of interest was reported by the authors.

## ETHICAL APPROVAL STATEMENT

Ethical approval was granted by the first author’s academic institution ethics committee. The study was approved for hosting by the Head of Psychology in Craigavon Area Hospital.

## PARTICIPANT CONSENT

All participants included in the current study provided written and audio consent for the inclusion of quotes in the current manuscript.

## Endnote

^1^
Please note that, unfortunately, a full discussion of EMDR is beyond the scope of this paper, which purported to primarily evaluate the compassion‐based elements of the model. For a comprehensive review of EMDR, see Shapiro (2014).
